# Induction of protein citrullination and auto-antibodies production in murine exposed to nickel nanomaterials

**DOI:** 10.1038/s41598-017-19068-1

**Published:** 2018-01-12

**Authors:** Bashir M. Mohamed, Noreen T. Boyle, Anja Schinwald, Bruno Murer, Ronan Ward, Omar K. Mahfoud, Tatsiana Rakovich, Kieran Crosbie-Staunton, Steven G. Gray, Ken Donaldson, Yuri Volkov, Adriele Prina-Mello

**Affiliations:** 1 Department of Clinical Medicine, Trinity College Dublin, Dublin, Ireland; 2 AMBER centre and CRANN Institute, Trinity College Dublin, Dublin, Ireland; 3 Department of Physiology, Trinity College Dublin, Dublin, Ireland; 4 MRC/University of Edinburgh, Centre for Inflammation Research, Queen’s Medical Research Institute, 47 Little France Crescent, Edinburgh, EH16 4TJ United Kingdom; 5 Ospedale dell’Angelo, Venice, Italy; 6 Department of Histopathology, St James’s Hospital, Dublin, Ireland; 7 Thoracic Oncology Research Group, St James’s Hospital, Dublin, Ireland

## Abstract

Citrullination, or the post-translational deimination of polypeptide-bound arginine, is involved in several pathological processes in the body, including autoimmunity and tumorigenesis. Recent studies have shown that nanomaterials can trigger protein citrullination, which might constitute a common pathogenic link to disease development. Here we demonstrated auto-antibody production in serum of nanomaterials-treated mice. Citrullination-associated phenomena and PAD levels were found to be elevated in nanomaterials -treated cell lines as well as in the spleen, kidneys and lymph nodes of mice, suggesting a systemic response to nanomaterials injection, and validated in human pleural and pericardial malignant mesothelioma (MM) samples. The observed systemic responses in mice exposed to nanomaterials support the evidence linking exposure to environmental factors with the development of autoimmunity responses and reinforces the need for comprehensive safety screening of nanomaterials. Furthermore, these nanomaterials induce pathological processes that mimic those observed in Pleural MM, and therefore require further investigations into their carcinogenicity.

## Introduction

Citrullination is involved in several pathological processes in the body, including autoimmunity and tumorigenesis. Citrullinated proteins are generated by a post-translational deimination or demethylimination of polypeptide-bound arginine by a family of Ca^2+^-dependent enzyme peptidylarginine deiminase (PAD)^1^. Several isotypes of PAD exist, each with different tissue distribution^2–5^. Citrullinated proteins are recognized as non-self-proteins, and can subsequently induce the development of autoimmune conditions^5–7^. The autoimmune response is linked to the pathogenesis of several diseases including rheumatoid arthritis (RA) multiple sclerosis (MS) and psoriasis^5–10^. It has been also reported that the levels of citrullinated proteins and PAD4 were elevated in patients with various cancers, such as oesophageal cancer, breast cancer and lung adenocarcinoma^11–15^. A more recent work by Jiang and co-workers reported that citrullinated proteins were detected in liver, breast, lung, ovarian, cervical, kidney, pancreatic and gastric tumour cell lines^15^.

It has also been shown that nanomaterials of diverse origin can trigger protein citrullination which might constitute a common pathogenic link to disease development^16,17^. Indeed, previous studies have associated human exposure to environmentally presented nano-size and ultra-fine materials with various pathologic processes, such as chronic inflammation, pneumonia, chronic obstructive pulmonary disease, autoimmune conditions^18–21^, pleural malignant mesothelioma (MM)^22–24^ and lung cancer^25^. Given these links, the increasing presence and application of manufactured nanomaterials has intensified investigations to assess their potential impact on human health. We hypothesised that engineered nickel nanomaterials which can mimic environmental fibre-and filamentous-type nanomaterials, trigger similar pathological responses.

Further to the previous reports^16,17^, this study uses well-characterised nanowires (NiNWs) of two defined lengths, to test the length-dependent pathogenicity hypothesis. A method of intrapleural injection of nanomaterials, described by Murphy *et al*.^26^, was used to measure organ specific response as well as the systemic response towards the nickel nanomaterials. This method is justified on the basis that a fraction of all inhaled particles and fibers that deposit in the peripheral lung translocate into the pleural space^27–31^. Subsequently, serum and tissue samples of NiNWs-treated mice were analysed for the presence of citrullinated proteins, the expression of PAD enzymes and formation auto-antibodies against citrullinated proteins. In parallel, we examined the expression profiles of PAD2, PAD4 and citrullinated proteins in human MM tissue samples.

Our results show that exposure to nickel nanomaterials increases the protein citrullination and the induction of PAD2 and PAD4 *in vitro* and *in vivo*. Furthermore, we report autoantibody production following nickel nanomaterials exposure in *in vivo* mouse model. The expression of PAD2 and PAD4 and the induction of protein citrullination were also demonstrated in pleural and pericardial MM tissue sections. Based on the comparison of the data collected in experimental and clinical samples from pleural MM patients with known prior asbestos exposure, we suggest that both asbestos fibres and nickel nanomaterials trigger elevation of PAD enzymes thereby altering citrullination pathways and resulting in the development of autoimmune responses.

## Results

### Nickel nanowires cause citrullination in human cells *in vitro*

**Figure 1:**
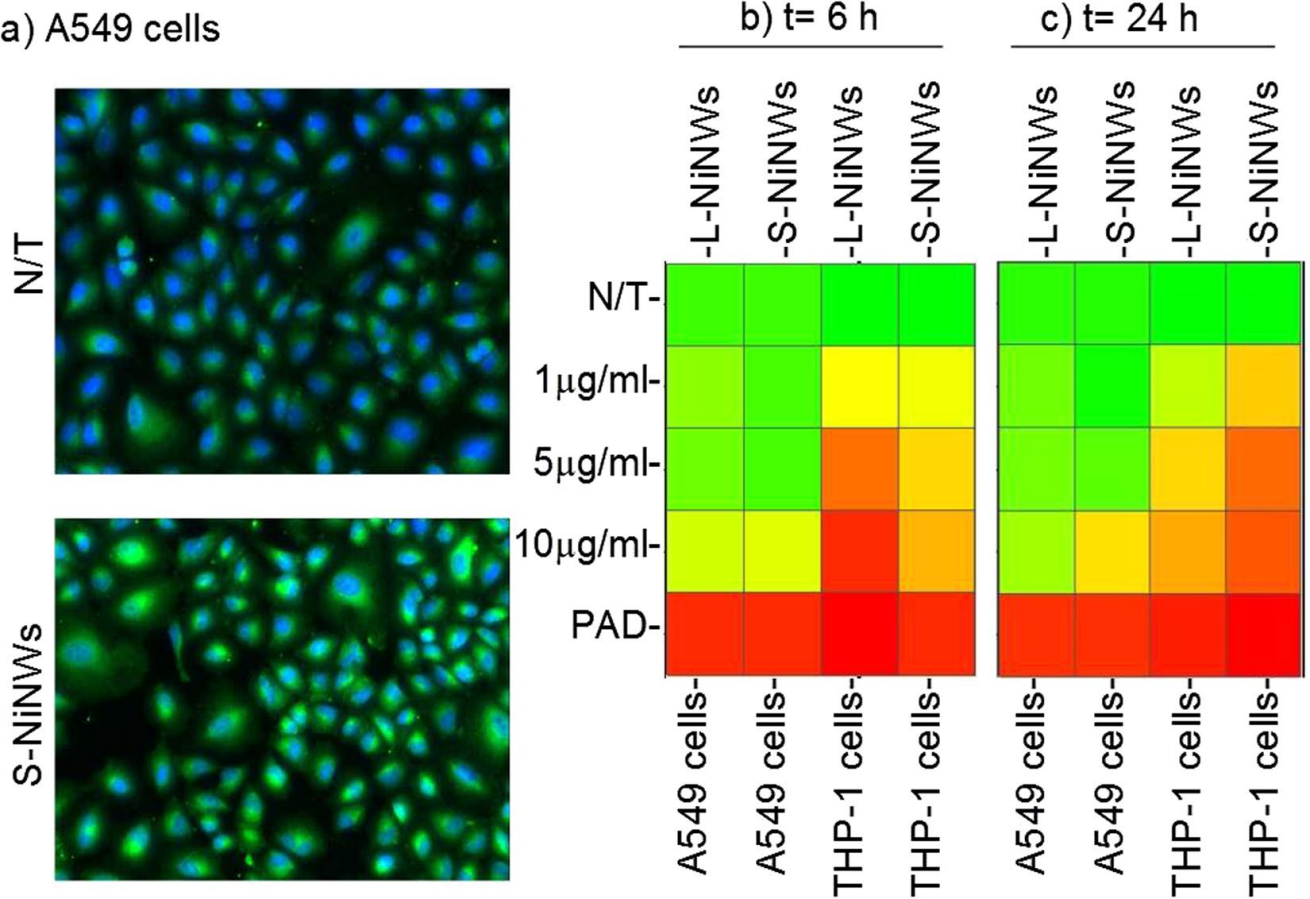
Citrullination levels in THP-1 and A549 cells exposed to nickel nanomaterials. (**a**) Representative fluorescent images of untreated controls (N/T), short (S)-NiNWs-treated A549 cells after 24 h exposure. Images show cytoplasmic citrullinated protein (green) and nuclei (blue). (**b** and **c**) Heatmaps reflect the citrullination levels indicated by colours ranging from red (≥75%) to yellow (50%) to green (≤25%) compared to negative control (N/T). PAD was used as positive control.

The differential ability of short and long nickel nanowires (NiNWs) to induce protein citrullination was studied in a human lung epithelial cell line A549 and a phagocytic cell line THP-1. Both cell lines showed induction of protein citrullination as examined by HCS (Fig. 1a) in significant dose-dependent manner with maximum effects seen at the highest dose (10 µg/ml) of NiNWs (Fig. 1b and c).

As previously shown by our group, increased calcium ion concentration and PAD enzymatic activity as key factors in the citrullination were induced by nanomaterials^16^. In agreement with this, the treatment of A549 and THP-1 cells with nanowires resulted in the significant induction of calcium ion accumulation in both cells (Supplementary Figure S1).

### Induction of protein citrullination in murine model

**Figure 2:**
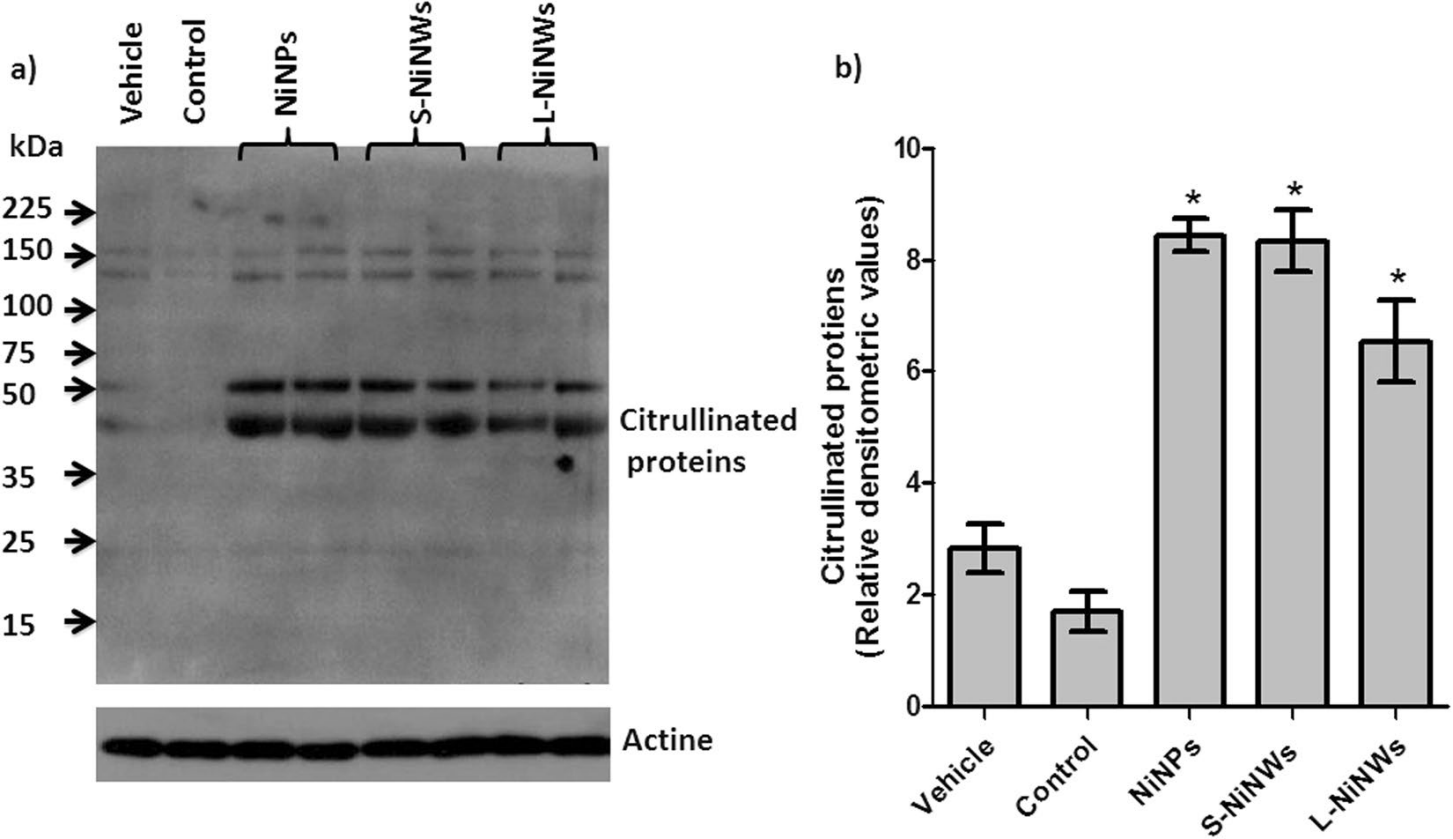
Induction of citrullinated proteins in mice sera following nickel nanomaterials exposure. (**a**) Serum samples from nickel nanomaterial-injected mice, vehicle control and untreated mice (N/T) were probed by immunoblotting technique for citrullinated proteins. (**b**) Densitometric quantitation of citrullinated protein bands indicated a significant increase in protein citrullination due to nickel nanomaterials exposure compared to vehicle and untreated controls. Data are representative of three independent experiments (mean ± S.E.). *p < 0.05 with respect to corresponding controls. Note: western blot gels were adapted to fit within composite image, full gel images are provided in Supplemental Information (Figure S2).

We further examined nickel nanomaterials’ potential to induce protein citrullination *in vivo*. We have detected distinct citrullinated protein bands (40–60 kDa) following exposure to S-NiNWs, L-NiNWs and NiNPs in mouse sera. Faint bands were detected at the level of 150 kDa in all experimental groups, including vehicle and untreated controls, which may represent naturally occurring citrullinated proteins (Fig. 2).

### Measurement of anti-CCP3 levels in mice following exposure to nickel nanomaterial

**Figure 3:**
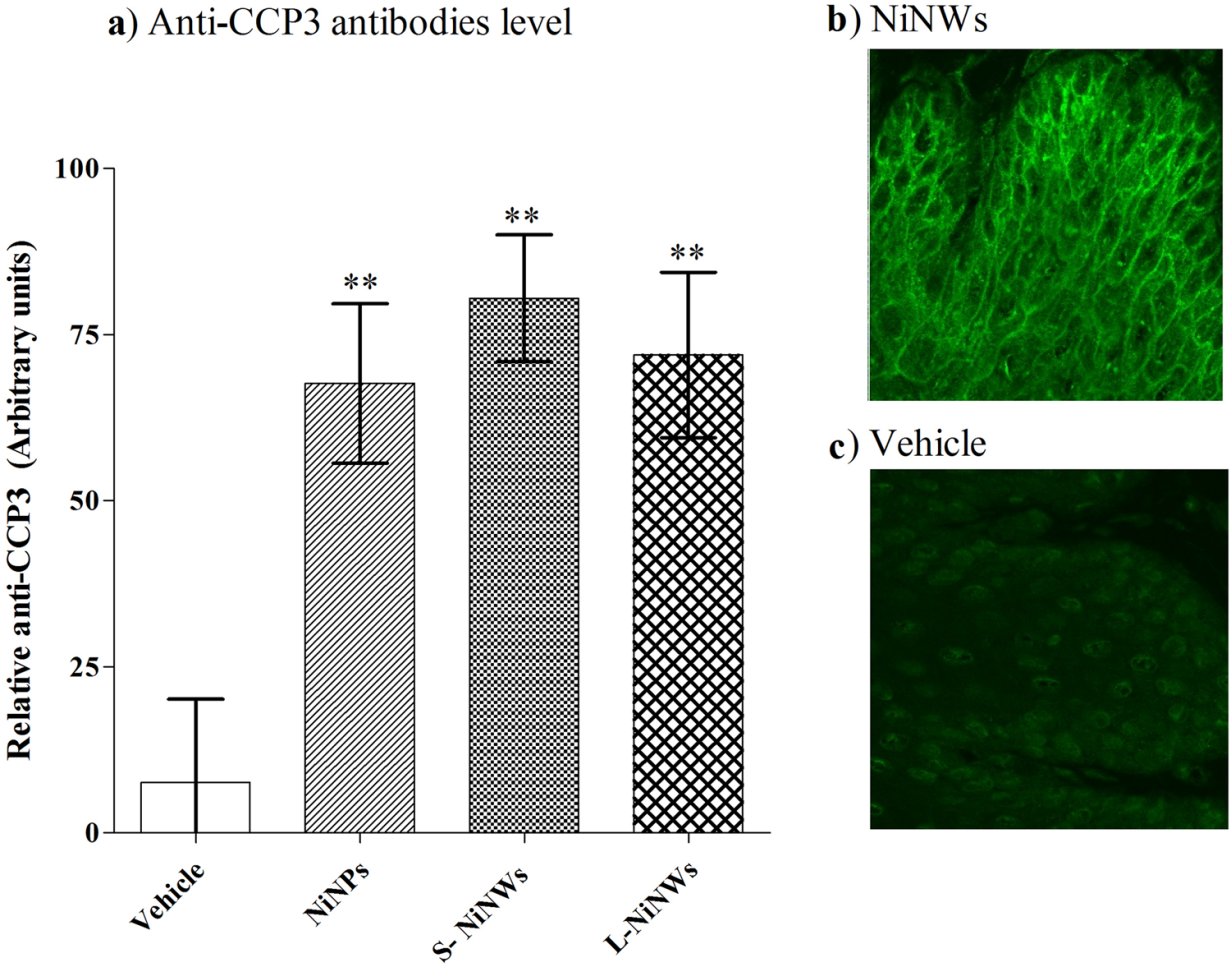
Detection of anti-CCP3 antibodies in murine sera. (**a**) Serum samples from nickel nanomaterial-injected and vehicle-injected mice were examined for anti-CCP3 antibodies by ELISA. (**b**,**c**) Reactivity of anti-citrulline antibody was confirmed by indirect immunofluorescence after incubating sera from mice exposed to S-NiNW and vehicle controls on oesophageal tissue sections.

Serum samples collected two weeks post exposure of mice to nickel nanomaterials showed significant levels of anti-CCP3 antibody by ELISA analysis (Fig. 3a). Using indirect immunofluorescence technique, the anti-filaggrin antibody reactivity was detected in these serum samples when applied to rat oesophagus. Comparison between S-NiNW and vehicle control provide evidence of such difference in reactivity (Fig. 3b,c).

### Citrullination level in murine lymphoid organs and kidneys

**Figure 4:**
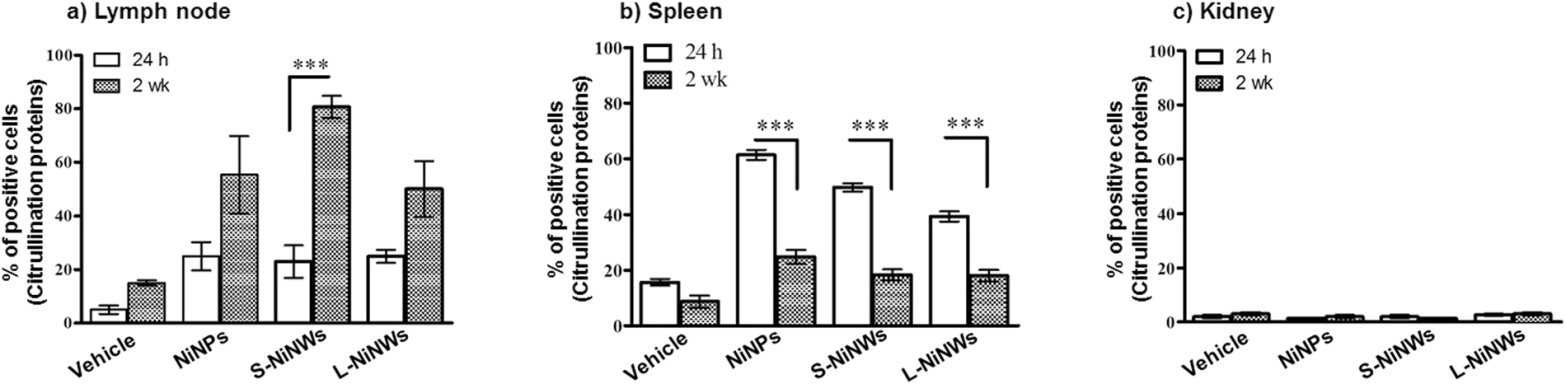
Immunohistochemical analysis of citrullinated proteins in murine lymphoid organs and kidneys. Mice were injected with PBS (vehicle) or NiNPs, S-NiNWs and L-NiNWs and the organs were analysed after 24 h and 2 weeks. LN and kidney tissue sections were stained with anti-citrulline antibody. Protein citrullination in LN (**a**) spleen (**b**) and kidney tissue sections (**c**) sections were compared to their group controls. *p*-values of <0.0001 are indicated by ***. Statistical analysis between the different experimental groups is reported in Table S1.

LN, spleens and kidneys of nickel nanomaterial-injected mice were tested for the incidence of intracellular protein citrullination. The data indicated a significant increase in the number of cells with citrullinated proteins in the LN tissue in mice treated with nickel nanomaterials, compared to vehicle control (Fig. 4a). Statistical analysis between the different experimental groups is reported in Table S1. Data accumulated from spleen tissue samples showed that the number of cells stained positive with anti-citrulline antibodies was elevated 24 h after exposure to nickel nanomaterials. This initial elevation was attenuated 2 weeks post-injection as shown in Fig. 4b. We did not detect protein citrullination in kidney tissue sections from nickel nanomaterials-injected mice (Fig. 4c).

### PAD2 expression in murine lymphoid organs and kidneys

**Figure 5:**
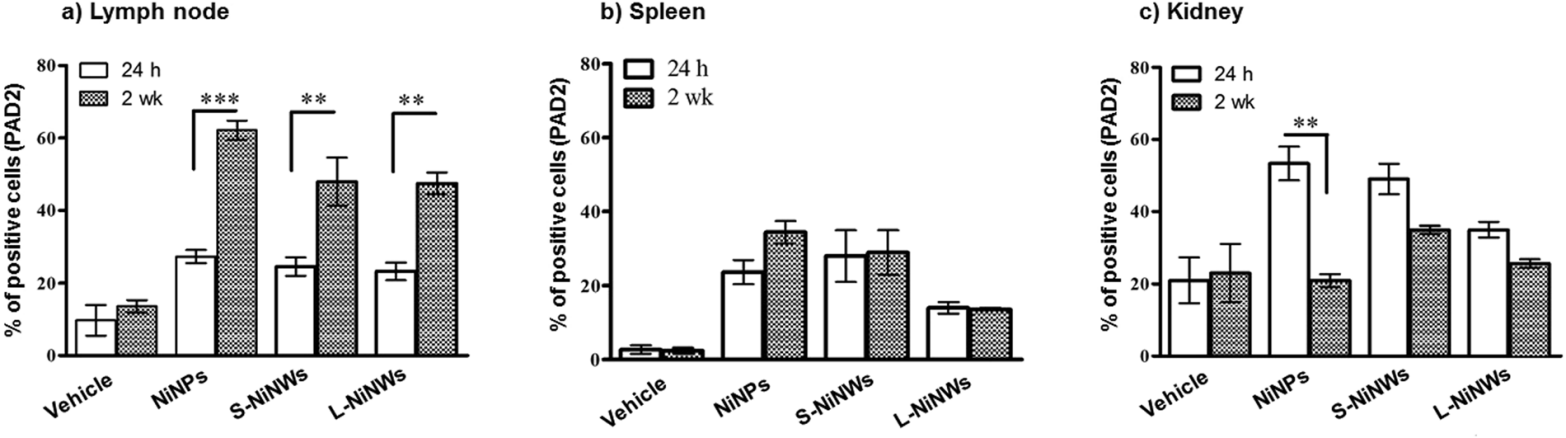
Immunohistochemical analysis of PAD2 expression in murine lymphoid organs and kidneys. Mice were injected with PBS (vehicle) or NiNPs, S-NiNWs and L-NiNWs for 24 h and 2 weeks. LN and kidney tissue sections were stained with anti-PAD2 antibody. The histograms shown the percentage of positively stained cells for PAD2 expression in LN (**a**), spleen (**b**), and kidney tissue sections (**c**) p-values of <0.0001 are indicated by ***. Statistical analysis between the different experimental groups is reported in Table S2.

Analysis of anti-PAD2 antibody staining in LN, spleen and kidney tissue sections showed an increase in the number of positive cells stained for PAD2 in all the nickel nanomaterial-treated groups. In LN tissue sections the percentage of cells expressing PAD2 was increased after 24 h versus control. These results were even more prominent at the 2-week time-point (Fig. 5a) due to the persistence of the nanomaterials within the organ. An increased number of cells with positive staining for PAD2 was observed in splenic tissue post nanomaterials exposure, however there was no significant difference between treatment groups at the two-time points (5 b). Analysis of kidney sections showed transient increase in PAD2-positive cells in nanomaterial-treated groups compared to vehicle alone (Fig. 5c). A significant reduction in the percentage of PAD2-positive cells in the NiNPs-treated group was recorded at 2 weeks. This difference could be associated with the shape of the nanomaterials, whereas spherical nanoparticles get a higher likelihood for kidney clearance.

### Expression of PAD4 in murine lymphoid organs and kidneys

**Figure 6:**
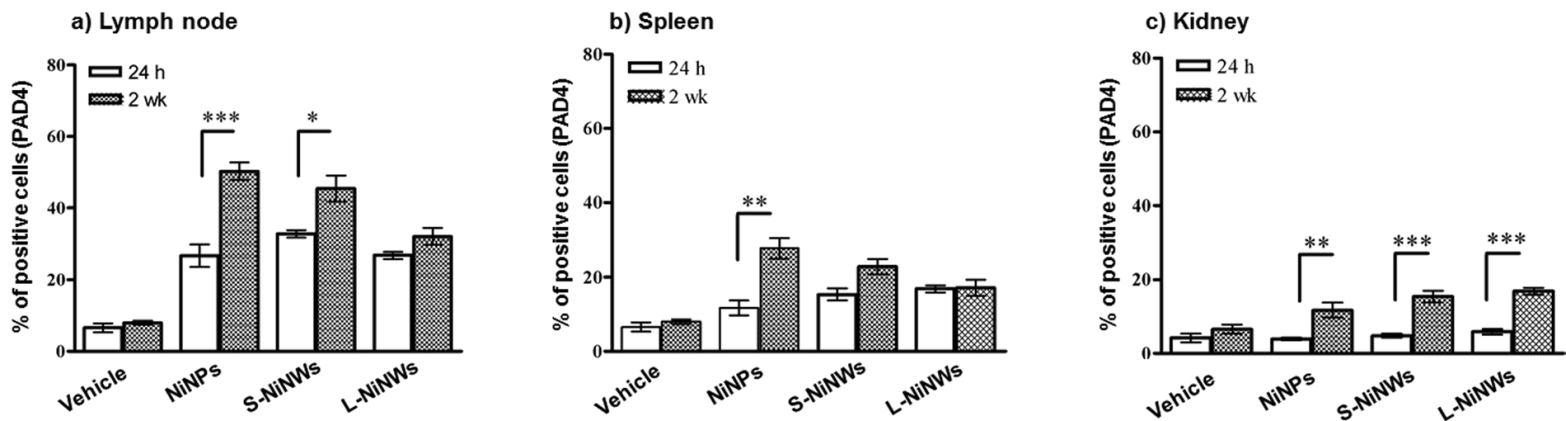
Immunohistochemical analysis of PAD4 expression in murine lymphoid organs and kidneys. Mice were injected with PBS (vehicle) or NiNPs, S-NiNWs and L-NiNWs for 24 h and 2 weeks. LN, spleen and kidney tissue sections were stained with anti-PAD4 antibody. PAD4 in LN (**a**) spleen (**b**) and kidney tissue (**c**) sections were compared to their group controls. p-values of <0.0001 are indicated by ***.

Analysis of anti-PAD4 antibody staining in LN, spleen and kidney tissue sections showed a significant increase of positive cells stained for PAD4 expression in all tissue sections of nickel nanomaterials-treated mice compared to vehicle (Fig. 6a,b). In LN tissue sections the percentage of cells expressing PAD4 was significantly increased after 24 h versus control. These results were even more prominent at the 2-week time-point (Fig. 6a). In the kidney tissue samples, PAD4-positive cells were detected only after 2-week exposure (Fig. 6c).

### Immunohistochemical analysis of PAD expression and citrullination in murine lymphoid tissue sections

**Figure 7:**
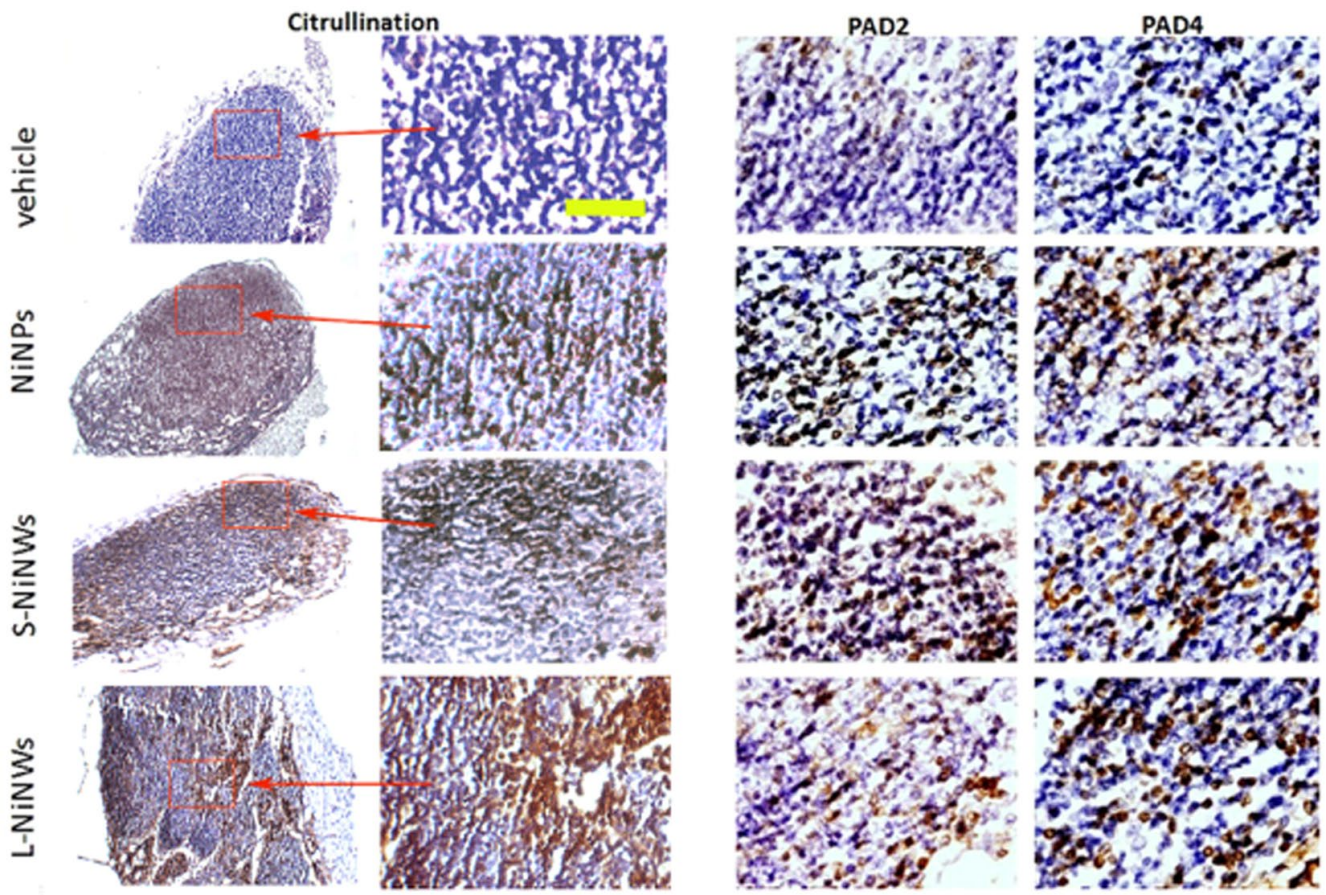
Immunohistochemistry of anti-citrulline, anti-PAD2 and PAD4 antibodies on murine lymph node (LN) tissue sections. Immunodetection of citrullinated proteins, PAD2 and PAD4 enzymes in murine LN tissue sections 2-week post treatment with nickel nanomaterials and vehicle controls. Brown staining demonstrates positive immunohistochemical reaction for citrulline, PAD2 and PAD4. First two panels from left (Citrulline) represent the corresponding microscopic fields at lower (10×) and higher (20×) magnification stained for citrullinated proteins, to illustrate the topography of positive staining within the LN. Red rectangles indicated by arrows outline the regions of interest magnified in the adjacent panel. Two panels on the right show representative topographically similar, but not identical microscopic fields stained for PAD2 and PAD4 enzymes, respectively. Scale bar (yellow): 100 μm.

**Figure 8:**
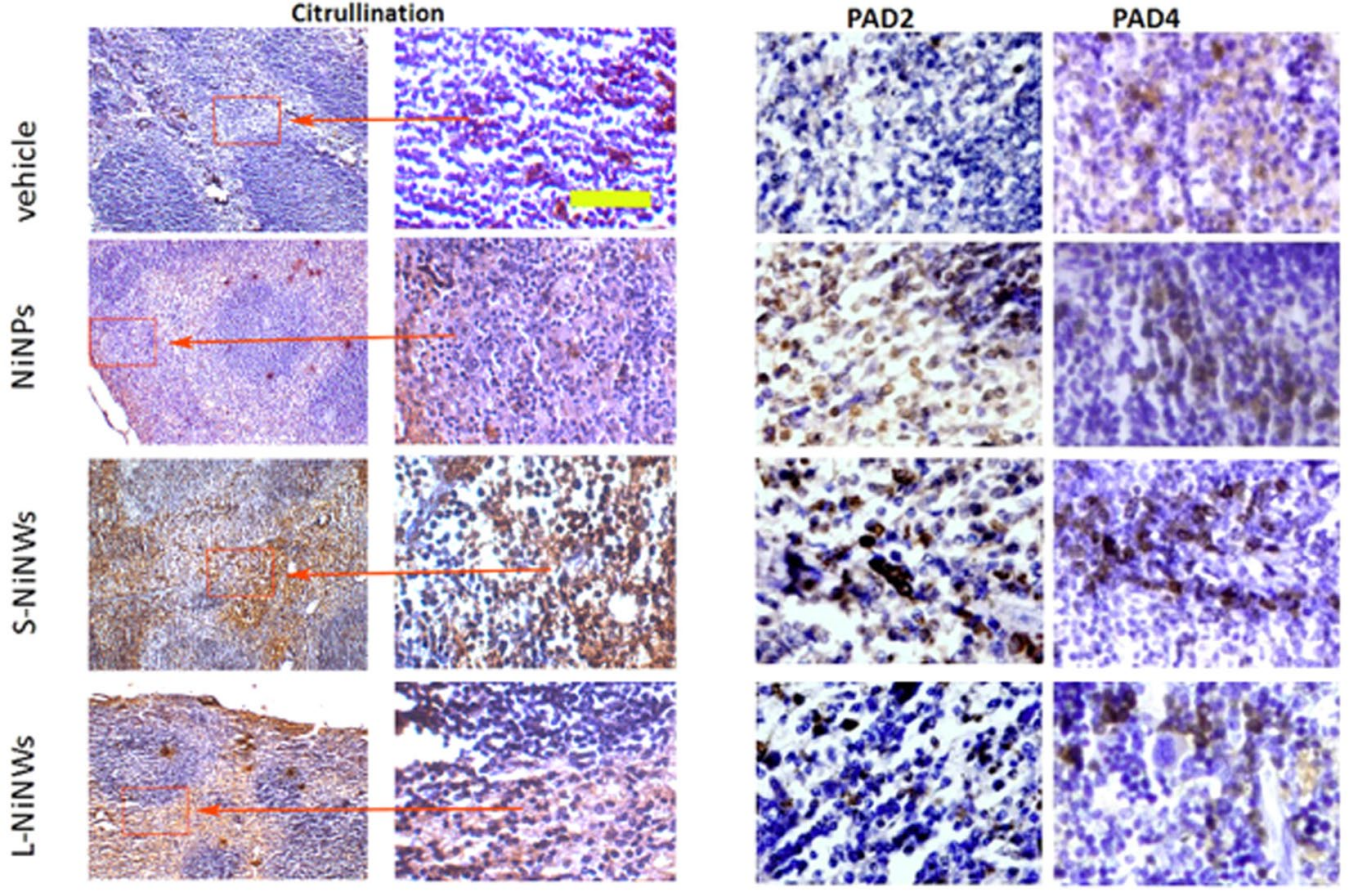
Immunohistochemistry of anti-citrulline, anti-PAD2 and PAD4 antibodies on murine spleen tissue sections. Immunodetection of citrullinated proteins, PAD2 and PAD4 enzymes in murine spleen tissue sections 2-week post exposure to nickel nanomaterials and vehicle controls. Brown staining demonstrates positive immunohistochemical reaction for citrulline, PAD2 and PAD4. First two panels from left (Citrulline) represent the corresponding microscopic fields at lower (10×) and higher (20×) magnification stained for citrullinated proteins, to illustrate the topography of positive staining within the spleen. Red rectangles indicated by arrows outline the regions of interest magnified in the adjacent panel. Two panels on the right show representative topographically similar, but not identical microscopic fields stained for PAD2 and PAD4 enzymes, respectively. Scale bar (yellow): 100 μm.

Tissues collected from the mice 24 h and 2 weeks post exposure to nanomaterials were immunohistohemically analysed for the PAD2, PAD4 expression and the presence of citrullinated proteins. Representative images of LN and spleen sections of 2-week post treatment mice are shown in Figs 7 and 8 respectively.

### Quantification of nanomaterial accumulation in murine lymphoid organs and kidneys

**Figure 9:**
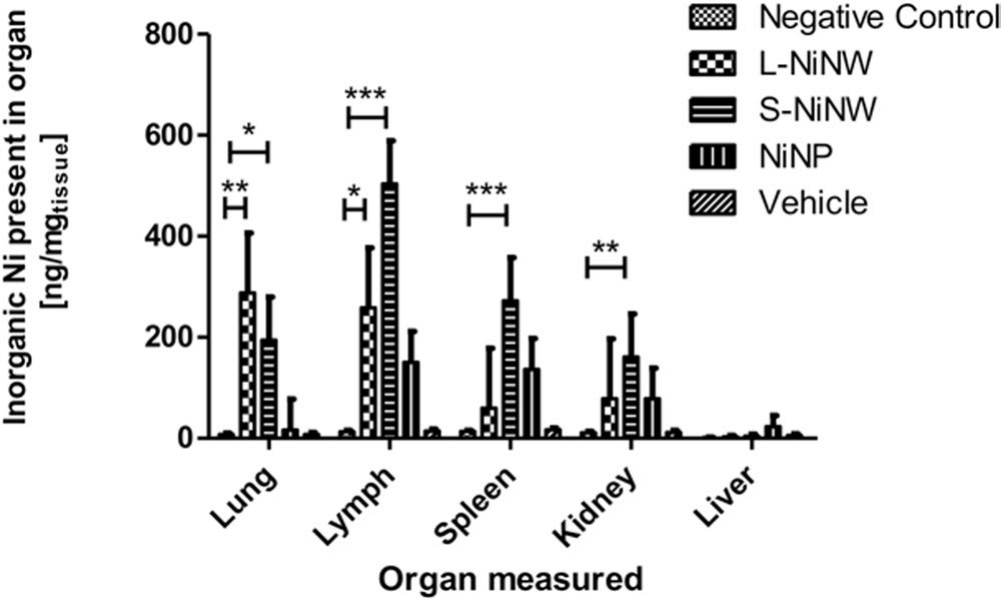
Quantification of inorganic nickel magnetic material in murine organs after 2-week exposure to nanomaterials (n = 3 animals). Comparison of total mass of nickel nanomaterial measured accumulated in each organ versus organ mass. Inorganic nickel content was measured by SQUID magnetometry as total amount of magnetic signal compared to vehicle. Data are shown as average ± standard error of the mean (n_repeats_ = 3). The symbols (*), (**) and (***) indicate statistical significant changes as compared to the values measured against negative control samples (p < 0.05, 0.01 and 0.001, respectively) (two-way ANOVA and Bonferroni post-test).

Quantification of inorganic nickel magnetic material present in mice organs was carried out in three animals after 2-week-nanomaterials exposure, as shown in Fig. 9. The results of the study were in agreement with previous work by Murphy *et al*. where they highlighted and quantified the presence of short and long nickel nanowire in mice mediastinal lymph nodes^26,32^. From the results presented, it can be seen that the accumulation of nickel nanomaterials in the murine organs is in agreement with the High Aspect Ratio Nanoparticles (HARN) paradigm since a high quantity of long nickel magnetic material (L-NiNW) is found in the lungs and similarly in the mediastinal lymph nodes; whereas for the short NiNW (S-NiNW) the accumulation is higher in the lymph nodes than the lung indicating a pleural barrier crossing, resulted to be statistically significant as shown Fig. 9. Similar trend of the accumulation of magnetic materials was also detected in spleen and kidneys whereas negligible amount was recorded in the liver, also found statistically significant for these organs in the case S-NiNW. Additional evidence on the preferential HARN accumulation was provided by the use of nickel nanoparticles as control material. There it can be seen that nickel nanoparticles are present in LN, spleen and kidneys in comparable amounts, while small amount was recorded in liver at suggesting that spherical particles can sustain longer circulation time compared to the fibre-like nanomaterials. Possible explanation lies on the different behaviour of the different HARN materials when exposed to the parabolic blood flow velocity thus where heavier and longer HARN would have shorter traveling distances compared to the shorter and lighter ones or spherical nanoparticles.

### Clinical samples of pleural and pericardial MM: induction of PAD2, PAD4 and protein citrullination expression

**Figure 10:**
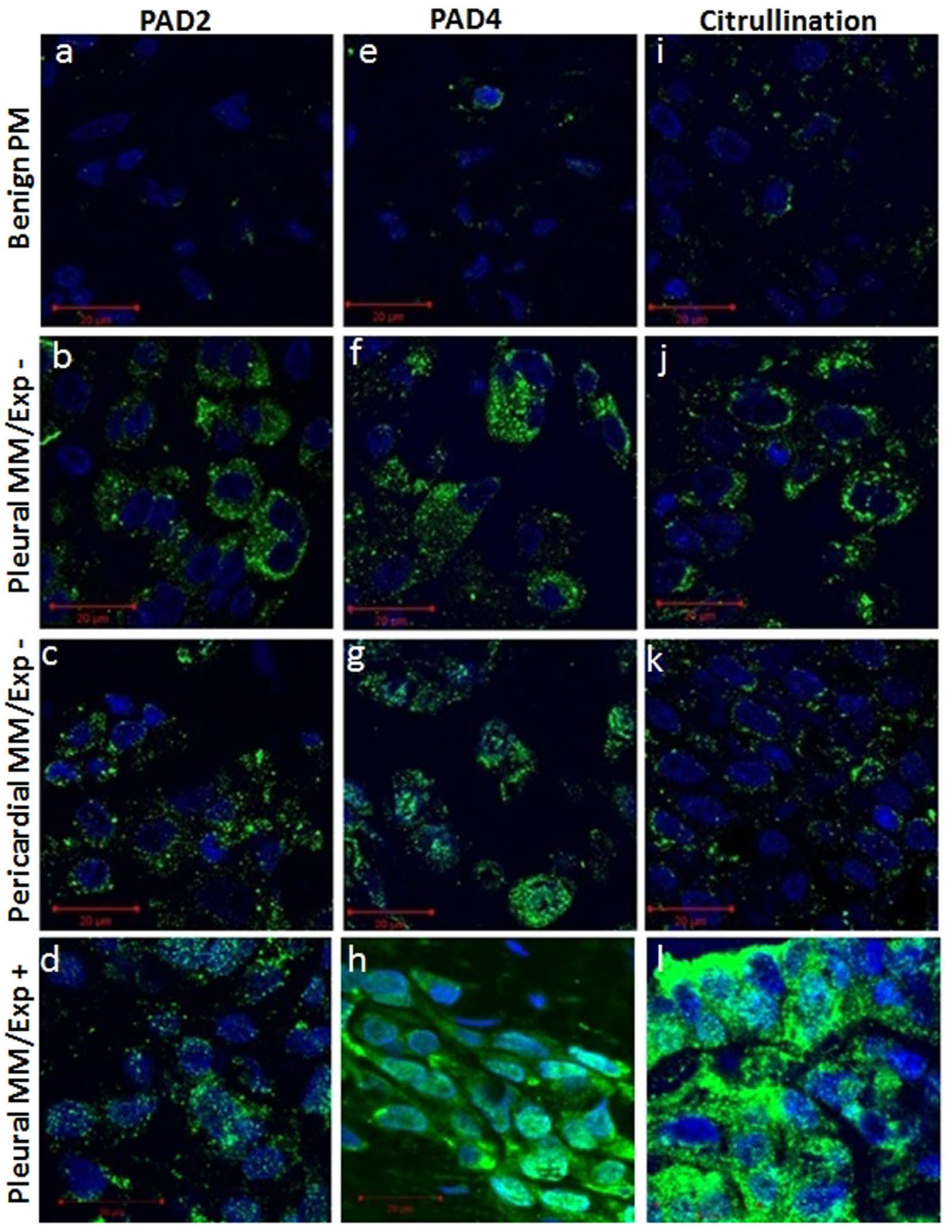
Immunodetection of PAD2, PAD4 expression and citrullinated proteins in human MM. abbreviations: MM/Exp+: Malignant mesothelioma from the patients with a known asbestos exposure, and MM/Exp −: Malignant mesothelioma from the patients without a history of asbestos exposure. PAD2 expression in tissue sections of (**a**) benign PM (**b**) and (**c**) pleural and pericardial MM/Exp− and (**d**) pleural MM/Exp+. PAD4 expression in tissue sections of (**e**) benign PM, (**f**) and (**g**) pleural, and pericardial MM/Exp− and (**h**) pleural MM/Exp+. Induction of protein citrullination in tissue sections of (**i**) benign PM, **(j**) and (**k**) pleural and pericardial MM/Exp− and (**l**) pleural MM/Exp+.

The measurements of PAD2 and PAD4 expression and protein citrullination in human pleural and pericardial MM with or without a history of patient’s exposure to asbestos (pleural MM/Exp+ and pleural and pericardial MM/Exp− and pleural) and benign Pleural Material (PM) was carried out using immunofluorescence method. As shown in Fig. 10, a very week staining of PAD2, PAD4 and protein citrullination was detected in tissue sections from benign PM. In contrast, increased staining of these markers was observed in pleural and pericardial MM/Exp− tissue sections. In addition, a strong staining of PAD4 and citrullinated proteins was found in tissue sections of pleural MM/Exp+. The finding of an increase of PAD2 and PAD4 expression and induction of protein citrullination in clinical samples of pleural MM with a known history of environmental exposure to asbestos indicates that such fibres can trigger similar pathogenetic mechanisms as engineered nickel nanowires.

## Discussion

The production of anti-citrullinated protein antibodies is almost 100% specific for patients with RA, indicating an important role of protein citrullination in the pathogenesis of RA^8^. However certain genetic predisposition has been identified such as HLA-DRB1^11,33^, however studies of external triggers that lead to disease manifestation have been inconclusive^8,34^. Furthermore, despite possible links between RA and environmentally imposed factors such as cigarette smoke, air pollution, and occupational exposure to nanomaterials such as silicon dioxide particles, the exact cellular events involved are still unclear^8,33–35^. Our group has previously demonstrated that nanomaterials of different origin are capable of promoting conversion of arginine residues to citrulline^16,17^, which may induce an autoimmune process and eventually lead to the development of RA. In this work, we tested the hypothesis that nickel nanomaterials of different geometrical size and aspect ratio could trigger a systemic citrullination response. Here we show for the first time, that *in vivo* exposure to nickel nanomaterials induces PAD2 and PAD4 expression, protein citrullination and production of auto-antibodies against citrullinated proteins.

The analysis of LN tissue sections from mice intrapleurally injected with nickel nanomaterials showed sustainable increase in the levels of citrullinated peptide residues. This increase was associated with higher PAD2 and PAD4 expression level. PAD2 and PAD4 have gained attention as potential candidates that drive the citrullination of self- antigens in RA^2,36^. Therefore, these results reinforce the concept that nanomaterial-mediated citrullination could be associated with inflammation or with the development of autoimmune conditions such as RA.

Distribution of nickel nanomaterials into the spleen is also possible through the bloodstream following intrapleural injection. Specifically, it has been previously shown that in the murine spleens, polystyrene nanoparticles accumulated in the marginal zones of lymphoid follicles due to the macrophage activity^37–39^. This is also coherent with previously published work where short nickel nanowires where found in lymphoid organs of mice^26^. In our study, a transient induction of intracellular protein citrullination was found in spleen tissue sections after exposure to nickel nanowires indicating the potential clearance of nanomaterials over time.

We found that prolonged exposure to nickel nanowires induce a transient expression of PAD2 and PAD4 in mouse kidneys, however no induction of protein citrullination was detected. The kidneys are predominantly responsible for the exclusion of metabolic waste such as urea and ammonia. The filtration barrier is mainly created by the glomerular visceral epithelial cells or podocytes. The podocyte forms the filtration barrier by synthesizing the glomerular basement membrane, elaborating filtration slits, and producing the slit diaphragms^40^. In addition, characteristic arrangement of cytoskeletal proteins such as vimentin in podocytes is required to sustain the complex structures^41^. Also, due to high blood supply level and ability to concentrate toxins, the kidneys could be potentially affected by any material that reaches the circulatory system.

Our findings of the transient expression PAD2 and PAD4 in the absence of induction of protein citrullination in the kidneys could be explained by relatively low enzymatic activity level in this organ insufficient to induce citrullination are of most abundant potential targets, such as for example podocyte vimentin. Other possibility is that the high Ca^2+^ levels known to be pre-requisite for PAD activation are not sustained in the kidneys.

Therefore, it would be reasonable to suggest that the primary citrullination phenomena induced by nanoparticles are developing in other organs and tissues rich in residential macrophages. This also could involve the uptake, transport and presentation of nanomaterials by antigen presented cells (APCs). Indeed, macrophages have previously been shown to phagocytose NiNPs and S-NiNWs, while L-NiNWs are only partially engulfed and cause frustrated phagocytosis, leading to inflammation promotion and influx of inflammatory cells^19^. Another possibility stems from studies in autophagy, where cellular components are degraded in an auto-catabolic mechanism as a means of self-clearance. Different nanoparticles have been observed to induce autophagy in various conditions, perhaps from oxidative stress or a need to eliminate foreign bodies from cells, similarly to viral infections^42–46^. Considering the possibility that autophagy in APCs mediates citrullination and antigen presentation to T cells, nanomaterial uptake by macrophages and/or dendritic cells could contribute to the pathogenesis of ACPA-positive RA through these mechanisms^47,48^. Our work further supports the differences between the phagocytic mechanisms induced by long versus short NiNW, as shown in Fig. 1 where the L-NiNWs are likely to exert immediate early-response and S-NiNWs might be involved in a delayed autophagy.

Our *in vivo* assays also demonstrated the presence of citrullination residues in mice sera following systemic administration of nickel nanomaterials. Of note anti-thrombin, an anti-coagulant with a molecular weight of 52 kDa, has been shown to become citrullinated, causing a loss of normal physiological function that may promote symptoms and pathology of RA through lowered thrombin inhibition and increased coagulation, inflammation and angiogenesis^14^. Thus, mouse anti-thrombin could be one of candidate citrullinated proteins found in sera and observed here in response to nickel nanomaterials. In certain diseases, 46 kDa keratin-18 is found in serum and could also be citrullinated either in tissue or in the circulation by PAD enzymes^49,50^. Our findings demonstrating the presence of citrullinated proteins in mouse sera (Fig. 2) following nickel nanowires exposure fully support these possibilities.

We demonstrate here the induction of citrulline-specific antigen autoantibodies following nickel nanomaterials exposure *in vivo*. Since the mice used in our experiments can develop spontaneous arthritis, it is conceivable that anti-CCP antibodies can appear as a part of the pathogenetic processes leading to autoimmune arthritis as previously suggested^51^.

Similarities between our experimental findings and data from human clinical samples were found in relation to the expression levels of PAD2 and PAD4 enzymes and downstream citrullinated proteins. These results highlight the possibility that engineered nanomaterials with high aspect ratio can trigger an autoimmune response and are in line with the fibre-pathogenicity paradigm^21,52,53^.

Our studies indicate that exposure to “naturally” occurring environmentally presented nanofibers, such as asbestos, induce PAD2, PAD4 activity and citrullination to the levels comparable to those registered following the exposure to well characterised-engineered nickel nanomaterials. It is well known that asbestos induces a chronic inflammation^54^. Of note, it has been demonstrated that nanomaterials with high aspect ratio are also able to cause chronic pleural inflammation^19,55,56^.

## Conclusions

The results of *in vivo* experiments demonstrated that engineered nickel nanowires were potent inducers of immune responses through the elevation of enzymatic activity of PAD enzymes, which in turn leads to the enhanced protein citrullination. In addition, we show for the first time the increased expression of PAD2, PAD4 and the induction of protein citrullination in clinical samples of pleural MM from the patients with known history of environmental exposure to asbestos, thereby suggesting a potential set of markers in identifying malignant mesothelioma patients at early stage of the disease.

Therefore, our overall findings provide fundamental evidence that engineered nickel nanowires evoke immune responses similar to those observed for environmental fibre-type nanomaterials such as asbestos, indicating that this may be a cause for serious concern in relation to the associated chronic inflammation and subsequent disease development.

Critically, as these nanomaterials mimic pathophysiological processes observed in malignant mesothelioma, further risk assessment studies and investigations regarding their carcinogenicity are of prime importance.

## Materials and Methods

### Nanomaterials

Engineered nickel nanowires of two discrete lengths were fabricated as previously reported^22,32,57^. Briefly, L-NiNWs and S-NiNWs were characterised by counting a minimum of 100 separate nanowires using scanning electron microscopy (Carl Zeiss Ultra Plus, UK) and by Nomarski technique. Average lengths of nanowires were found to be 4.3 ± 1.0 µm (further referred to as short nanowires, S-NiNW), and 24.0 ± 7.2 µm (further referred as long nanowire, L-NiNW); this in accordance with previously published work^20,32^. Commercially available nickel nanoparticles were also used in the tests as an alternative type of nickel-based nanomaterial (Nanostructured & Amorphous Materials Inc., TX, USA). These had a mean dry diameter of 15.0 ± 5.0 nm, (as declared by manufacturer’s description and further refereed to NiNP) and could be readily dispersed in saline (Sigma-Aldrich, Poole, UK) with a resulting hydrodynamic diameter of 57.0 ± 10.0 nm as reported in previously published work^58^.

### Cell culture

Human lung epithelial cell line A549 and a phagocytic cell line THP-1 (ATCC, Manassas, VA, USA) were cultured in DMEM and RPMI 1640 medium, respectively. Both types of media were supplemented with 10% (v/v) foetal bovine serum, 1% (v/v) L-glutamine/ penicillin/streptomycin. Cells were grown in a humidified incubator at 37 °C in 5% CO_2_. THP-1 cells were treated with phorbol 12-myristate 13-acetate (25 ng/ml) before experimentation.

### *In vivo* studies

The *in vivo* work was carried out in accordance to the FELASA guidelines and recommendations by fully trained staff holding a valid UK Home Office personal licence. Ethical use of animal and were approved by the UK Home Office under approved project licence in accordance to the international FELASA guidelines. 40 female C57BL/6 mice (Harlan, UK) were housed in a group size of five in standard caging with sawdust bedding within a pathogen-free Home Office approved facility as previously described^19^. Mice were maintained on a normal 12-hour light and dark cycle. The animals were allowed 7 days to acclimatise prior to study commencement, after which groups of three animals were injected intrapleurally with 0.5 ml of 100 g/ml (50 g/animal) of NiNPs, S-NiNWs, L-NiNWs or 0.5 ml of 0.5% phosphate buffered saline (PBS) as a vehicle; in accordance to previously published work^19^. Animals were immediately placed back into their cages and monitored to ensure normal behaviour and for signs of distress or issues. The animals were sacrificed at 24 h and 2 weeks, then blood samples were collected and tissue samples from liver, mediastinal lymph nodes (LN), spleen and kidneys were harvested, fixed in formalin and paraffin blocks were prepared and processed for immunohistochemistry staining. From every second animal, isolated organs were used for inorganic magnetic nickel detection by SQUID magnetometry. All *in vivo* work was carried out by staff holding a valid UK Home Office personal licence under a Home Office approved project licence. Untreated mice (N/T) were also used as control.

### Patient’s samples

To facilitate identification of PAD2, PAD4 expression and the induction of protein citrullination in MM, four patients with known history of asbestos exposure (MM/Exp+) were included in this study. All patient samples were acquired in accordance to the Declaration of Helsinki based on the ethical principles for medical research involving human subjects. Patients gave full and informed consent and immunohistochemistry was conducted on all FFPE archival material as per St James’s Hospital Ethical Approval 041018/8804. These patients were identified from the archival pathology files of the Pathology Unit of the Regional Hospital of Mestre Venice, Italy. All were diagnosed with pleural MM based on World Health Organization criteria. The pleural MM was confirmed in all patients by clinical, morphological and immunohistochemistry data. The tissue samples were formaldehyde fixed and paraffin embedded. In addition, we also included a tissue microarray (TMA) containing 4 cases diagnosed with MM without any history of patient’s exposure to asbestos (MM/Exp−) and 2 cases with benign pleural tissue (US Biomax, Inc.).

### Antibodies and reagents

Rabbit polyclonal antibodies to citrulline, PAD2 and PAD4 antibodies and FITC-linked goat anti-rabbit antibodies were purchased from Abcam (Abcam plc, Cambridge, UK). Horseradish peroxidase conjugated anti-rabbit IgG and anti-mouse IgG antibodies were obtained from Dako A/S (Glostrup, Denmark). Peroxidase complex detection method was purchased from Novocastra Laboratories Ltd., UK.

### *In vitro* analysis of cellular citrullinated proteins

Cells were seeded in 96-well plates (1 × 10^4^ cells/well), exposed to various concentrations of nanomaterials for multiple time points and fixed using 3% paraformaldehyde as described^16^^,^^17^. They were immuno-stained for citrullinated proteins and high content screening assays (HCSA) were performed on IN Cell Analyser 1000 equipped with IN Cell Investigator software (GE Healthcare, Buckinghamshire, UK). Images were acquired and analysed as previously described^16,17^.

### SDS-PAGE and Western Blot analysis of citrullinated proteins

As previously described^16^, cell lysates (60 µg) were boiled with 1×NUPAGE® LDS sample buffer and resolved on a pre-cast 4–12% Bis-Tris NUPAGE® SDS-PAGE gel using NUPAGE® MOPS buffer. The resolved proteins were transferred to a polyvinylidene fluoride (PVDF) membrane by the wet transfer method using the XCell II™ Blot Module for 2 h. PVDF membrane was blocked in 5% non-fat dry milk in TBST (150 mM NaCl, 0.1% (v/v) Tween 20, 20 mM Tris-HCl) for 1 h at room temperature and incubated with the primary antibodies (specific to polymer chains of the amino acid citrulline) and then with a horseradish peroxidase conjugated secondary antibody. The bands were detected with the using LUMINATA Western HRP Substrate (EMD Millipore Corporation, Billerica, MA, USA) enhanced chemiluminescent detection system and subsequent exposure to Kodak light-sensitive film. Densitometric analyses of the western blots were performed by using GeneTools software (Syngene). The relative values of the samples were determined by giving an arbitrary value of 1.0 to the respective control samples of each experiment.

### *In vivo* detection of antibodies against citrullinated proteins

Sera from each mouse were collected from the tail vein on the initial day of nanomaterials exposure and 7 days thereafter. On the day of sacrifice, sera were collected by cardiac puncture. Anti-cyclic citrullinated proteins (anti-CCP3) antibodies were determined using the Quanta Lite Anti-CCP3 IgG ELISA test (third generation peptides; Inova Diagnostics, Inc. San Diego, USA) following manufacturers’ protocol. Mouse sera were diluted 1:80 in sample diluent, and the secondary antibody was replaced with rabbit anti-mouse (CALTAG Laboratories, Buckingham, UK) diluted 1:1,000 in PBS.

### Immunofluorescent staining

Anti-filaggrin antibody reactivity was determined by indirect immunofluorescence. Undiluted sera or sera diluted 1:10 in PBS from mice post exposed to the nickel nanomaterials, vehicles, and untreated mice were applied to rat oesophageal sections (Scimedx Corporation, Denville, NJ 07834, USA) for 1 hour at room temperature with humidity. The slides were then washed 3 times for 5 minutes in PBS. FITC conjugated goat anti-mouse IgG (BD Biosciences-Pharmingen) was applied for 1 hour at room temperature in a humid environment. The slides were again washed 3 times for 5 minutes in PBS then Dako mounting medium and cover slip placed on the slide. The samples were visualized using confocal microscopy (LSM Meta-510, Carl Zeiss, Axiovert, Germany).

### Immunohistochemistry staining

The tissue sections preparation including preservation and fixation was performed as previously described^59^. Immunohistochemical analysis for the presence of citrullinated proteins, PAD2 and PAD4 expression was conducted on 5 µm tissue sections of LN, kidneys, liver and spleen using the peroxidase complex detection procedure^60–62^.

### Confocal Microscopy

5 µm tissue sections of pleural and pericardial malignant mesothelioma with no information of asbestos exposure (pleural MM and pericardial MM/Exp−), tissue sections from patients with known exposure to asbestos (pleural MM/Exp+) and tissue section of normal pleural tissue were mounted on glass slides and stained with anit-PAD2, anti-PAD4 and anti-citrullination antibodies using immunofluorescence staining methods. Cell nuclei were visualised with Hoechst 33342 (Sigma-Aldrich Corporation, St Louis USA) as previously described^63^. Slides were sealed with Dako Fluorescent Mounting Medium (Dako Diagnostics Ireland Ltd). Confocal images of immunostained tissue section were taken with the Zeiss LSM Meta-510 confocal microscope (Carl Zeiss, Axiovert, Germany). Two channel qualitative imaging was carried out using the 63× oil immersion objective.

### Total quantification of the inorganic magnetic nickel nanowire material

Quantification of the inorganic magnetic nickel nanowire material present in different organs explanted from exposed mice was carried out by assessing the total magnetic signal collected from each organ sample. Magnetization measurements were made at room temperature using a Quantum Design MPMS XL SQUID magnetometer (Quantum Design, Inc., CA, USA). Same volume of organ samples was loaded into gel capsules mounted in a non-magnetic straw. All measurements were made using a standard second-order gradiometer, around the maximum slope position of the second derivative curve, with no auto-tracking of the actual sample position. Prior and post measurement empty and full capsules were weighted (Model AX26DR, Mettler Toledo, USA) in order to then calculate the total magnetic nickel nanowire material mass against the full sample mass. Total mass of magnetic nickel nanowire material was calculated from the magnetisation measurement carried out compared to the magnetisation saturation of the same weight mass of magnetic bulk nickel powder, which as a magnetisation saturation of 55.4 A·m^2^/kg.

### Statistical analysis

Response of each cell type to various nanoparticles was analysed by *2-way ANOVA* with Bonferroni post-test analysis. A *p*-value of <0.05 was considered to be statistically significant (GraphPad Prism 4, GraphPad Inc., USA). KNIME data exploration platform and the screening module HiTS were used to visualize the data. Protein citrullination level induced by the nanomaterials was normalized to positive controls. Scoring was done by using Z score of the normalized values and summarized as described in previous work^64^. Heatmap-type graphical illustration in a colorimetric gradient table format was adopted as the most suitable schematic representation.

## Electronic supplementary material

Supplemental Information
