# Supplementary material for: Induction of protein citrullination and auto-antibodies production in murine exposed to nickel nanomaterials
Source: Sci Rep. 2018 Jan 12;8:679. doi: 10.1038/s41598-017-19068-1 (PMC5766588; doi:10.1038/s41598-017-19068-1)
Supplement: Supplementary file 1 — Supplemental Information [file 41598_2017_19068_MOESM1_ESM.pdf]

# Induction of protein citrullination and auto-antibodies production in murine exposed to nickel nanomaterials

Bashir M. Mohamed[1,7], Noreen T. Boyle[2,3], Anja Schinwald[4], Bruno Murer[5], Ronan Ward[6] Omar K Mahfoud[1], Tatsiana Rakovich[1], Kieran Crosbie-Staunton[1], Steven G. Gray[7], Ken Donaldson[4], Yuri Volkov[1,2] Adriele Prina-Mello[1,2]

[1]Department of Clinical Medicine, [2]AMBER centre and CRANN Institute, and [3]Department of Physiology, Trinity College Dublin, Ireland,

[4]MRC/University of Edinburgh, Centre for Inflammation Research, Queen's Medical Research Institute, 47 Little France Crescent, Edinburgh EH16 4TJ, United Kingdom

[5]Ospedale dell'Angelo, Venice, Italy,

[6]Department of Histopathology and [7]Thoracic Oncology Research Group, St James's Hospital, Dublin, Ireland.

# Supplemental Information

Corresponding author:

Adriele Prina-Mello, PhD

Trinity Translational Medicine Institute (TTMI), School of Medicine, AMBER centre, CRANN Institute, Trinity College Dublin, the University of Dublin

Postal address: Room 0.75, Institute of Molecular Medicine Trinity Centre for Health Sciences James's street Dublin 8, Ireland

T: +353 1 896 3259 / 3087

E: prinamea@tcd.ie

## Nickel nanowires cause citrullination in human cells *in vitro*

Additional experiments confirmed the increased calcium ion concentration, as already observed in our in vitro models following exposure to nanowires [1]. A549 and THP-1 cells were exposed to S-NiNWs and L-NiNWs (10 μg/ml) in the presence or absence of a $Ca^{2+}$ Channel Blocker (CCB) verapamil (10 μM). (A & B) Intracellular levels of free $Ca^{2+}$ were measured and plotted as RFU. (C & D) Verapamil treated or untreated cells (NT) were exposed to S-NiNWs and L-NiNWs for 24 h, PAD4 expression was analysed and plotted as relative fluorescence units (RFU). Data are mean ± standard error of mean of three independent experiments performed in triplicates.

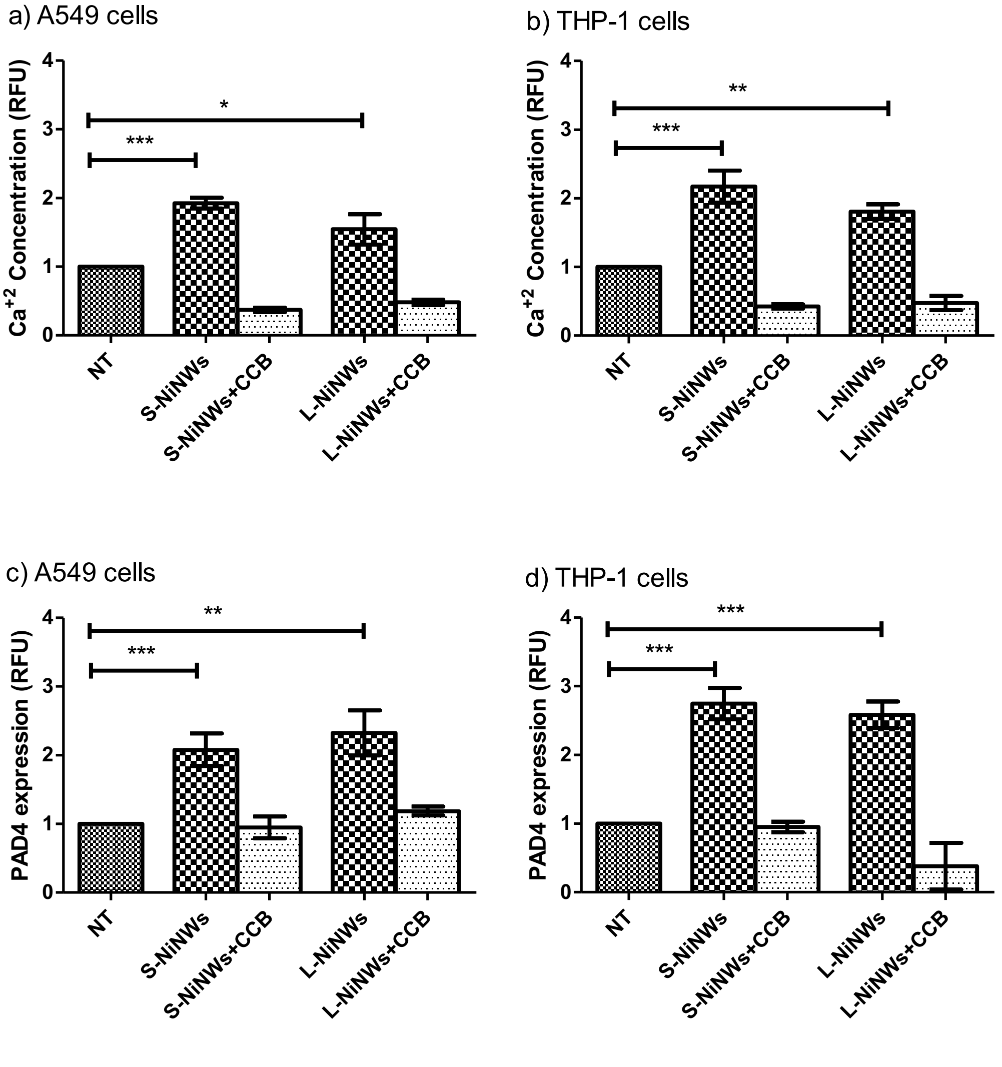


**Figure S1**. Measurements of intracellular $Ca^{2+}$ levels in human lung epithelial cells line (A549 cells) and a phagocytic cell line (THP-1 cells) exposed to nickel nanomaterials and the effect of blocking $Ca^{2+}$ channels on PAD expression.

**SDS-PAGE and Western Blot Analysis of Citrullinated Proteins.**

As from materials and methods, and from previously reported by some of the authors in a previous publication[1] cell lysates (60 µg) were boiled with 1x NUPAGE® LDS sample buffer and resolved on a pre-cast 4-12% Bis-Tris NUPAGE® SDS-PAGE gel using NUPAGE® MOPS buffer. The resolved proteins were transferred to a polyvinylidene fluoride (PVDF) membrane by the wet transfer method using the XCell II™ Blot Module for 2 h. PVDF membrane was blocked in 5% non-fat dry milk in TBST (150 mM NaCl, 0.1% (v/v) Tween 20, 20 mM Tris-HCl) for 1 h at room temperature and incubated with the primary antibodies (specific to polymer chains of the amino acid citrulline) and then with a horseradish peroxidase conjugated secondary antibody. The bands were detected with the using LuminataTM Western HRP Substrate (EMD Millipore Corporation, Billerica, MA, USA) enhanced chemiluminescent detection system and subsequent exposure to Kodak light-sensitive film. Densitometric analyses of the western blots were performed by using GeneTools software (Syngene).

Western blot gels are reported below for the analysis of citrullinated proteins bands from serum samples due to nickel nanomaterials exposure compared to vehicle and untreated controls, Figure S2.

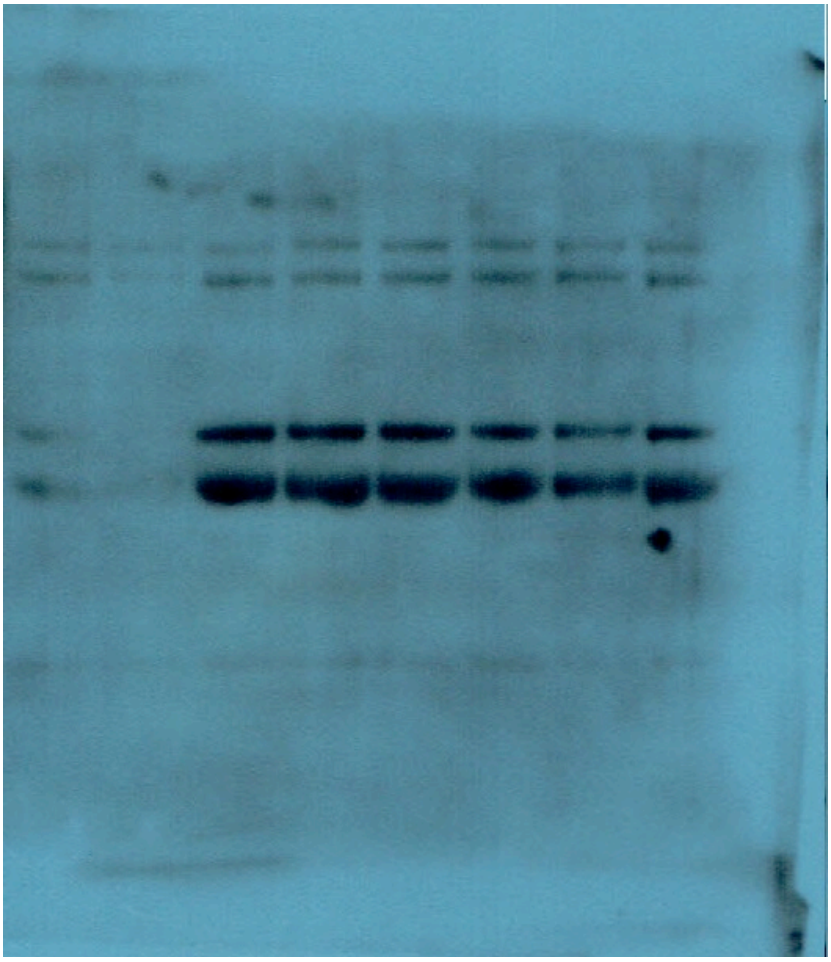

**Figure S2:** Western blot showing citrullinated proteins bands from serum samples (from left to right): Vehicle control and untreated mice (N/T), exposed mice with nickel nanoparticles, exposed mice with short nickel nanowires, exposed mice with long nickel nanowires

**Table S1 a,b.c:** CITRULLINATION comparative statistical analysis of NiNPs, S-NiNWs and L-NiNWs (24h and 2wks) versus their Vehicle control. Statistical significance expressed in p-value. p-value Not significant (NS) > 0.05 * < 0.05, ** < 0.01 and *** < 0.001.

| **a) Lymph node** | 24h | 2wks |
|---|---|---|
| Vehicle vs NiNPs | NS | * |
| Vehicle vs S-NiNWs | NS | *** |
| Vehicle vs L-NiNWs | NS | NS |
| NiNPs vs S-NiNWs | NS | NS |
| NiNPs vs L-NiNWs | NS | NS |
| S-NiNWs vs L-NiNWs | NS | NS |

| **b) Spleen** | 24h | 2wks |
|---|---|---|
| Vehicle vs NiNPs | *** | *** |
| Vehicle vs S-NiNWs | *** | NS |
| Vehicle vs L-NiNWs | *** | NS |
| NiNPs vs S-NiNWs | * | NS |
| NiNPs vs L-NiNWs | *** | NS |
| S-NiNWs vs L-NiNWs | * | NS |

| **c) kidneys** | 24h | 2wks |
|---|---|---|
| Vehicle vs NiNPs | NS | NS |
| Vehicle vs S-NiNWs | NS | NS |
| Vehicle vs L-NiNWs | NS | NS |
| NiNPs vs S-NiNWs | NS | NS |
| NiNPs vs L-NiNWs | NS | NS |
| S-NiNWs vs L-NiNWs | NS | NS |

**Table S2 a,b.c:** PAD2 expression comparative statistical analysis of NiNPs, S-NiNWs and L-NiNWs (24h and 2wks) versus their Vehicle control. Statistical significance expressed in p-value. p-value Not significant (NS) > 0.05 * < 0.05, ** < 0.01 and *** < 0.001.

| **a) Lymph node** | 24h | 2wks |
|---|---|---|
| Vehicle vs NiNPs | NS | *** |
| Vehicle vs S-NiNWs | NS | *** |
| Vehicle vs L-NiNWs | NS | *** |
| NiNPs vs S-NiNWs | Ns | NS |
| NiNPs vs L-NiNWs | NS | NS |
| S-NiNWs vs L-NiNWs | NS | NS |

| **b) Spleen** | 24h | 2wks |
|---|---|---|
| Vehicle vs NiNPs | * | *** |
| Vehicle vs S-NiNWs | ** | ** |
| Vehicle vs L-NiNWs | NS | NS |
| NiNPs vs S-NiNWs | NS | NS |
| NiNPs vs L-NiNWs | NS | * |
| S-NiNWs vs L-NiNWs | NS | NS |

| **c) kidneys** | 24h | 2wks |
|---|---|---|
| Vehicle vs NiNPs | ** | NS |
| Vehicle vs S-NiNWs | * | NS |
| Vehicle vs L-NiNWs | NS | NS |
| NiNPs vs S-NiNWs | NS | NS |
| NiNPs vs L-NiNWs | NS | NS |
| S-NiNWs vs L-NiNWs | NS | NS |

Reference


1 Mohamed, B. M. *et al.* Citrullination of proteins: a common post-translational modification pathway induced by different nanoparticles in vitro and in vivo. *Nanomedicine* **7**, 1181-1195, doi:10.2217/nnm.11.177 (2012).
